# Bacterial Endophytic Communities in the Grapevine Depend on Pest Management

**DOI:** 10.1371/journal.pone.0112763

**Published:** 2014-11-11

**Authors:** Andrea Campisano, Livio Antonielli, Michael Pancher, Sohail Yousaf, Massimo Pindo, Ilaria Pertot

**Affiliations:** 1 Research and Innovation Centre, Fondazione Edmund Mach (FEM), S. Michele all'Adige (TN), Italy; 2 Austrian Institute of Technology GmbH, Department of Health & Environment, Bioresources Unit, Tulln, Austria; 3 Department of Environmental Sciences, Quaid-i-Azam University, Islamabad, Pakistan; Wageningen University and Research Centre, Netherlands

## Abstract

Microbial plant endophytes are receiving ever-increasing attention as a result of compelling evidence regarding functional interaction with the host plant. Microbial communities in plants were recently reported to be influenced by numerous environmental and anthropogenic factors, including soil and pest management. In this study we used automated ribosomal intergenic spacer analysis (ARISA) fingerprinting and pyrosequencing of 16S rDNA to assess the effect of organic production and integrated pest management (IPM) on bacterial endophytic communities in two widespread grapevines cultivars (Merlot and Chardonnay). High levels of the dominant *Ralstonia*, *Burkholderia* and *Pseudomonas* genera were detected in all the samples We found differences in the composition of endophytic communities in grapevines cultivated using organic production and IPM. Operational taxonomic units (OTUs) assigned to the *Mesorhizobium*, *Caulobacter* and *Staphylococcus* genera were relatively more abundant in plants from organic vineyards, while *Ralstonia*, *Burkholderia* and *Stenotrophomonas* were more abundant in grapevines from IPM vineyards. Minor differences in bacterial endophytic communities were also found in the grapevines of the two cultivars.

## Introduction

Endophytic microorganisms are found in virtually all plants studied. Interest in endophytes has soared in recent years because of growing evidence that they may play a vital role in plant health, growth and overall physiology [1]–[3]. Close interaction with the host has also been considered increasingly important due to their possible use in agriculture as new biocontrol agents and biofertilisers [4]. Recent technical advances in DNA and RNA sequencing technologies have radically changed the approach to the study of microbial communities, their assembly and functioning [5], [6]. The amount of sequence data that can be produced at relatively low cost has improved our insight into plant-associated microbial communities [7], but posed new challenges regarding the treatment and analysis of these large datasets, as the exploration of entire plant-associated microbial communities has become possible [8], [9]. This new interest has started to shed light on how management practices and plant physiology affect plant-associated microbiota, for example [10], [11]. The effects of crop and pest management, namely integrated pest management (IPM) and organic production, on crop and soil microbial communities has been partly investigated [12]–[14]. A deeper understanding of how plant protection affects endophytic microorganisms is required to shape agricultural policy in the future, since they may impact crop quality and health. Both organic production and IPM aim to reduce/avoid the use of chemical pesticides in agriculture and therefore their residues in food crops (thus minimising their impact on the environment). In Europe, organic production is regulated by Council Regulation (EC) No 834/2007 [15] which sets out the principles for production and in particular establishes a ban on chemically synthesised input. Integrated pest management is not yet regulated in Europe, however the general principles of IPM are listed in Annex III of Directive 2009/128/EC [16], to achieve the sustainable use of chemical pesticides.

The extent to which the genetic diversity of grapevines cultivars influences the assembly of endophytic communities is unknown. In the roots of the annual potato plant, bacteria showed a strong correlation with the cultivar [9]. In grape must, the cultivar appeared to drive community composition, possibly through specific interactions between the berry and its surface microbiota [22]. We previously showed minor differences in grapevine fungal endophytes in the two Merlot and Chardonnay cvs. [12] using ARISA.

Full sequencing of a plant's endophytic metagenome has occasionally been achieved by employing complex procedures [17] or extensive deep genome sequencing [18], but it remains a challenging task, primarily because it entails separation of the plant host genome from its metagenome. A relatively easier approach (as compared to plant-associated metagenome sequencing) is analysis of the composition of microbial endophytic communities using DNA-dependent methods involving PCR and amplicon analysis. Although taxonomic composition analysis alone cannot describe the functioning of microbial communities, a shift in community composition is considered a clear sign of community restructuring [19], which may in turn reflect a functional modification.

Grapevine-associated microbiota, especially bacteria, has been investigated in recent studies, highlighting some aspects underlying community composition, assembly and variability [20]–[23]. Our aim in this study was to address the impact of two pest management types (organic production and IPM) on the composition of endophytic microbial communities. We adopted a combination of DNA-dependent approaches (ARISA and 454 pyrosequencing), in order to explore the effects of such practices on the composition of native microbial populations in two widespread *Vitis vinifera* L cultivars (Merlot and Chardonnay). Although the grapevine has received more attention than other crops, only a small number of studies have addressed bacterial diversity in perennial crops and woody plants [20], [22], [24], [25]. These studies either attempt full-scale analysis of the microbiome associated with the plant [20], [25], linking grapevine *terroir* and wine characteristics to associated microbiota [22], or investigate the relationship between pathogen infection and grapevine endophytes [19]. Understanding the effect of human activities on crops and the associated microbial communities is crucial, both because of the ecological implications and to provide the background for shaping future pest management policy.

## Materials and Methods

### Ethics statement

No specific permits were required for any of the locations (coordinates available in Table 1) where the plants were sampled. Within the Province of Trentino the Fondazione Edmund Mach has access to the vineyards used for this study and can collect biological samples. All the land is privately owned. The land at Navicello (Rovereto) is owned by Fondazione Edmund Mach. No protected species were sampled. No animals were involved in this study.

**Table 1:** sample codes and their origin.

Area	Location	Cultivar	Pest Management	Sample name
	45°43′35.14″N	Chardonnay	Organic	CO2A
**A**	10°56′55.99″E	Merlot	Organic	MO2A
Avio-Ala	45°43′28.97″N	Chardonnay	Integrated	CI13A
	10°56′44.06″E	Merlot	Integrated	MI13A
	46° 1′36.40″N	Chardonnay	Organic	CO4B
**B**	10°57′38.33″E	Merlot	Organic	MO4B
Pergolese	46° 1′41.91″N	Chardonnay	Integrated	CI19B
	10°57′26.97″E	Merlot	Integrated	MI19B
	45°54′48.86″N	Chardonnay	Organic	CO8C
**C**	11° 0′58.21″E	Merlot	Organic	MO8C
Noarna	45°54′13.14″N11° 1′22.37″E	Chardonnay	Integrated	CI17C
	45°54′17.57″N11° 0′52.56″E	Merlot	Integrated	MI16C
	45°53′16.49″N	Chardonnay	Organic	CO9D
**D**	11° 0′6.39″E	Merlot	Organic	MO9D
Isera	45°53′3.96″N11° 0′4.76″E	Chardonnay	Integrated	CI14D
	45°53′9.59″N11° 0′13.42″E	Merlot	Integrated	MI15D
	45°52′46.30″N	Chardonnay	Organic	CO12G
**G**	11° 1′15.98″E	Merlot	Organic	MO12G
Navicello	45°52′33.36″N	Chardonnay	Integrated	CI18G
	11° 1′2.87″E	Merlot	Integrated	MI18G

Products, active ingredients and target microorganisms used in IPM and organic production in the area and during the season of the study.

### Plant material and sampling

We sampled grapevines in five locations that served as replicates (http://goo.gl/maps/7AI7j, the exact coordinates being listed in Table 1). At each location (where a homogenous climate was assumed) four vineyards were chosen, one vineyard for each of the four treatments: organic Merlot, IPM Merlot, organic Chardonnay and IPM Chardonnay (Table 1). Four plants, representing the replicates, were randomly sampled in each vineyard. As we sampled adult plants growing in vineyards dedicated to the production of wine grapes, the experimental design was not the typical split plot, but rather made use of separate, existing vineyards. The grapevines used in this work were of similar size and age. This could represent a source of variation in the composition of microbial communities in vineyards that we could not separate in our assessment. All sampling took place simultaneously during the autumn, between October 27 and November 11, 2010. The impact of the environment on the variability of samples from the different sampling areas was minimised by following strict selection criteria: plant material was sampled only from lateral stems (or shoots) of field grapevines in a restricted geographic region (Trentino, northern Italy), with medium sandy, calcareous soils [26] characterised by a humid, temperate, oceanic climate typical of alpine foothill areas, with maximum rainfall in spring and autumn [27]. Grapevines were grafted onto SO4 or Kober 5BB rootstocks. Plant material was stored and pre-processed as described previously [12].

### DNA extraction, handling and amplification

DNA was extracted from surface-disinfected and aseptically peeled grapevine branches, as described previously [12]. Plant shoots were surface-disinfected by a succession of 2 min immersions, conducted under sterile laminar air flow, in 90% ethanol, 2.5% sodium hypochlorite solution, 70% ethanol and sterile distilled water. A sterile scalpel was used to aseptically remove the plant periderm. Plant material was then pulverised in sterile steel jars using liquid nitrogen and a mixer-mill. Following pulverisation, total DNA was extracted using the FastDNA spin kit for soil and a FastPrep-24 mixer (MP Biomedical, USA) according to standard manufacturer protocols. The concentration of extracted DNAs was estimated using a NanoDrop 8000 (Thermo Scientific, USA). PCR for bacterial ARISA (B-ARISA) was performed using FAM-labelled primers 2234C/3126T as previously described [28] and resolved by capillary electrophoresis on a ABI Prism 3130xl Genetic analyzer, equipped with a 50 cm capillary array filled with POP 7™ polymer (Applied Biosystems, USA). The electropherograms were analysed using Gene Mapper 4.0 and with peaks normalization inside the experiment. The fluorescence threshold was set to 50 relative fluorescence units (RFU). Peak binning was set to 1.5 bp and manual correction was applied where peak shifts occurred as described previously [29]. Operational taxonomic unit (OTU) frequency in each vineyard was scored on a zero to four index, considering presence/absence in each of four replicate plants, as reported previously [12].

### Pyrosequencing of the 16S rDNA gene

Samples from Isera were used for 454 pyrosequencing of the bacterial 16S rDNA gene amplicons. This location was chosen because the number of B-ARISA markers shown represented the overall distribution well. PCR was performed using High Fidelity FastStart DNA polymerase (Roche, USA) and the 799F (AACMGGATTAGATACCCK) and 1520R (AAGGAGGTGATCCAGCCGCA) universal primers with 454 adaptors and a sample-specific barcode on the forward primer. These primers allow selective amplification of bacterial DNA, targeting 16S rDNA hypervariable regions v5–v9 [30] without amplification of plastid DNA [31]. The PCR mix was prepared according to the manufacturer's instructions and included 5% DMSO and 50 ng of template DNA. Thirty cycles of PCR were carried out according to the manufacturer's instructions (as detailed for fragments below 3 kb) with the following parameters: the annealing temperature was 58°C, the elongation time was 1 min and the final elongation time was 7 min. The PCR product was separated on 1% agarose gel and gel-purified using Invitrogen PureLink (Invitrogen, USA). Amplicons were quantitated with quantitative PCR using the Library quantification kit – Roche 454 titanium (KAPA Biosystems, USA) and pooled in equimolar ratio in the final amplicon library. Pyrosequencing was carried out on the Roche GS FLX+ system using the new XL+ chemistry dedicated to long reads of up to 800 bp, following the manufacturer's recommendations.

### Data analysis

Raw SFF (standard flowgram format) files were pre-processed in Mothur [32] and quality was checked in PRINSEQ [33]. Data from read sequences, quality, flows and ancillary metadata were analysed using the Quantitative Insights Into Microbial Ecology (QIIME) pipeline [34]. Quality filtering consisted of discarding reads <200 nt and >1000 nt, excluding homopolymer runs >6 nt and ambiguous bases >6, accepting 1 barcode correction and 2 primer mismatches. A value of 25 was considered as the minimum average Phred quality score allowed in reads in a sliding window of 50 nt. Denoising was performed using the built-in Denoiser algorithm [35] and chimera removal and OTU picking accomplished with USEARCH, considering a pairwise identity percentage of 0.97. [36], [37]. Singleton OTUs were removed for statistical analysis. Taxonomy assignment was performed employing the naïve Bayesian RDP classifier with a minimum confidence of 0.8 [38] against the Greengenes database, October 2012 release [39].

To correct for sampling bias, a randomly selected subset based on the number of sequences in the poorest sample (2808 reads) was calculated in QIIME and used for further analyses.

All data were tested for normality (Shapiro-Wilk) and a log (x)+1 transformation (for x>0) was applied to meet the criteria for normal distribution [40], [41]. Since the data did not pass the normality test in both cases, nonparametric tests based on permutations were applied for further analysis.

OTU-based analysis was performed on both the ARISA and pyrotag-based datasets to calculate richness and diversity. Richness indices, Chao1 estimator [42] and abundance-based coverage estimator (ACE) [43] were calculated to estimate the number of observed OTUs present in the samples. The diversity within each individual sample was estimated using the nonparametric Shannon diversity index [44] and Simpson's diversity index [45]. Richness and diversity were estimated using the phyloseq R package (1.7.24) [46]. A confirmatory nonparametric permutation test was calculated in QIIME with 1,000 Monte Carlo permutations in order to compare alpha diversity values in agricultural practices and between cultivars.

Multivariate analysis of community structure and diversity was performed on the ARISA- and pyrotag-based datasets using: 1) unconstrained ordination offered by Principal Coordinate Analysis (PCoA) [47], 2) constrained multidimensional scaling using Canonical Analysis of Principal Coordinates (CAP) [48], 3) a permutation test for assessing the significance of the constraints and permutational multivariate analysis of variance (PERMANOVA), 4) indicator value analysis of taxa (for the pyrotag-based dataset only, where taxa could be identified) associated with the grouping factors used as constraints [49], [50]. The differences between bacterial communities were investigated using the Bray-Curtis dissimilarity distance [51] and the ordination methods applied to the matrix calculated in this way. All the ordination analyses were computed and plotted in phyloseq (points 1 and 2). The significance of the cultivar and the pest management grouping factors used as constraints in the CAP was assessed via the permutation test [52] in the vegan R package (2.0–10). The null hypothesis of no differences between *a priori* defined groups (*i.e.* assuming no constraints, as for the PCoA) was investigated using the PERMANOVA approach [53], implemented in vegan as the ADONIS function and applied to the Bray-Curtis dissimilarity distance. Indicator value analysis was calculated using the indicspecies R package [54], with the aim of identifying taxa associated with the Chardonnay rather than the Merlot cultivar or with IPM rather than organic production.

A comparison between ARISA and 16S rDNA pyrotags ordinations (for both PCoA and CAP) was carried out by means of procrustes analysis (PROTEST). PROTEST was performed on PCoA and CAP ordinations in order to evaluate the significance of the assessment of beta-diversity originating from B-ARISA and 16S rDNA.

Correlation between distance matrices was also calculated using the Mantel test [55]. A weighted Unifrac dissimilarity matrix [56] was also calculated in QIIME for the 16S rDNA-pyrotags, jackknifing (100 reiterations) read abundance data at the deepest level possible (2808 reads) (data not shown). To generate the phylogenetic tree on which the UniFrac distance was based, the representative sequences for each cluster (OTU) were aligned using PyNAST [57] against the Greengenes database (core set aligned sequences v.2010) and the tree was generated using FastTree [58]. The phylogenetic tree with the relative abundance of each OTU in the four treatments was then visualised using the iTOL tool [59]. Cytoscape [60] was used to visualise a network in which the samples and OTUs are the nodes. The network layout was edge-weighted spring embedded, based on eweights [61], the distance between nodes being optimised depending on eweight (which is a proxy measure for OTU abundance per sample, and ultimately for sample relatedness). The network is shown using two different sets of colours to highlight the interaction of the grapevine cultivar with pest management and of pest management with the taxonomy (at phylum level) of the associated OTUs.

## Results

### B-ARISA fingerprinting

B-ARISA fingerprinting of endophytic communities in the grapevine detected 251 OTUs, ranging from 200 to 1600 bp in length. The most frequent OTUs were found in the size range of 590–640 bp, with the single most frequent OTU corresponding to a peak with an estimated size of 633 bp.

Plants of the Chardonnay and Merlot cultivars showed an average ± standard deviation of 62.8±47 (n = 10) and 67.3±40 (n = 10) OTUs respectively. As regards pest management, in IPM grapevines an average of 28±22 (n = 10) OTUs was observed, whereas in organically produced grapevines, the average was 102.1±17 (n = 10). The nonparametric permutation test (after 1,000 Monte Carlo iterations) reported no significant differences between cultivars (n = 10) for any of the indices calculated above. Differences in pest management values were instead shown to be significant (p<0.01) for each of the diversity and richness indices.

**Figure 1:**
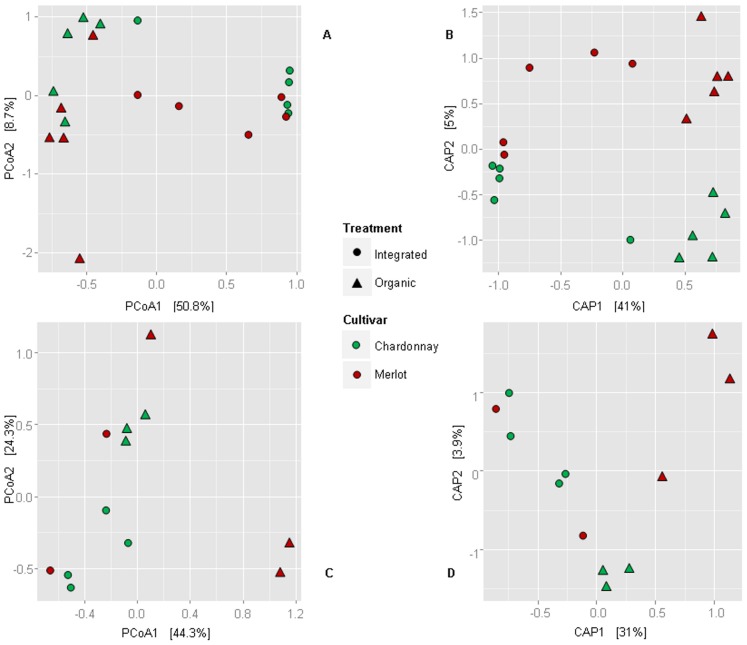
Multivariate analysis of beta-diversity: two-dimensional scatter plots of endophytic community composition in vineyards. A: PCoA of B-ARISA markers; B: CAP of B-ARISA markers; C: PCoA of 16S rDNA data; D: CAP of 16S rDNA markers. Ellipses and triangles represent samples from IPM and organic vineyards respectively; samples taken from Merlot and Chardonnay cvs are shown in red and green respectively.

B-ARISA marker distribution was investigated using PCoA and CAP to visualise differences between groups. Samples from organic and IPM farms were clearly separated along the main coordinate, explaining 50.8% and 41% of the variance in PCoA and CAP respectively (Fig. 1A and 1B). CAP analysis also suggested that Merlot- and Chardonnay- specific endophytic communities were somewhat different, although to a lesser extent than those of organic and IPM vineyards (the second CAP coordinate explaining only 5% of the variance). Interestingly, statistical treatment of B-ARISA results indicated that the difference between endophytic communities from organic and IPM vineyards was highly significant (*p*<0.001) when analysed using a permutation test for CAP scale after 9999 reiterations, while the difference between endophytic communities found in Merlot and Chardonnay was not statistically significant. Permutational multivariate analysis of variances (9999 permutations) applied on the distance matrix previously used for the ordinations, confirmed that only the difference between pest management types was significant (p = 0.0001).

### Roche 454 pyrosequencing

Pyrosequencing yielded 105,283 raw pyrotag reads distributed among 12 samples. Four samples were represented by a low number of reads and were thus removed from the statistical analysis (one sample each was removed from organic Chardonnay and organic Merlot, while two samples were removed from IPM Merlot). After quality filtering and chimera removing, a total of 74,966 high-quality sequences remained for community analysis. This corresponds to an average of 6,247±3,243 pyrotags per sample, with an average read length of 613 bp and a min and max of 200 and 781 bp.

**Figure 2:**
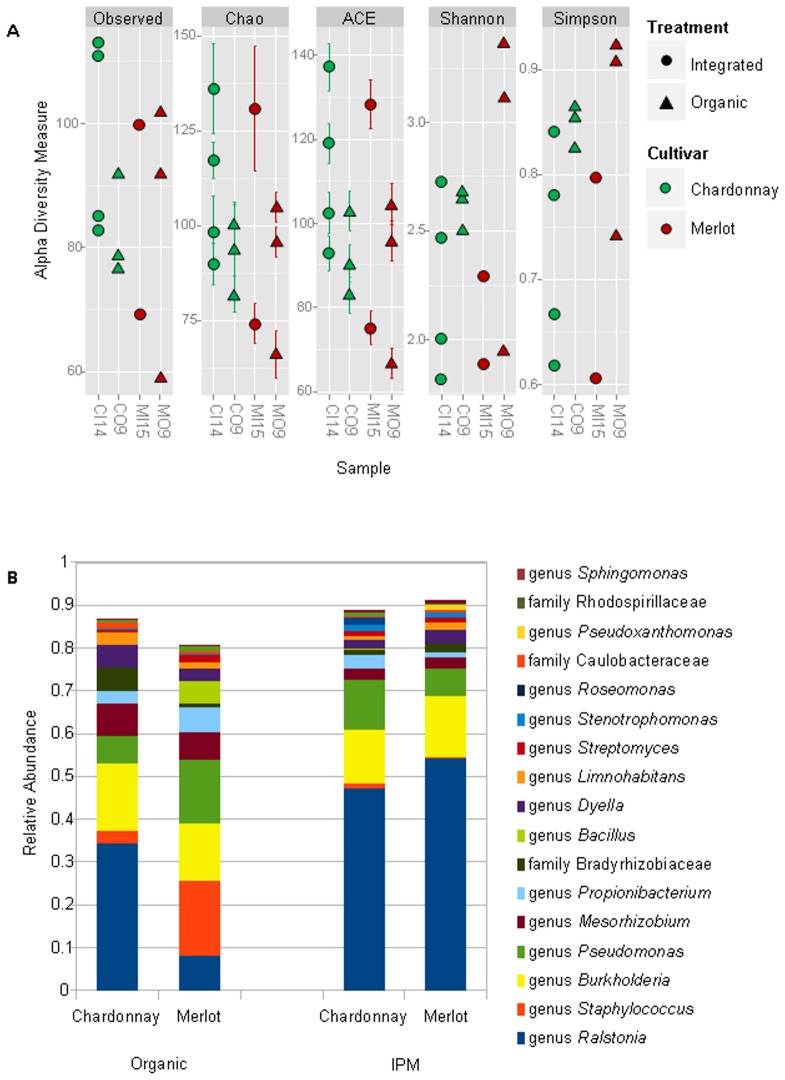
Microbial community analysis plots based on 16S rDNA pyrosequencing. A: alpha diversity metrics based on observed OTUs, richness (Chao's richness and Abundance-Based Coverage estimators) and diversity (Shannon's and Simpson's diversity indices) B: histogram representing taxonomic composition and relative abundance (over 2%) at family and *genus* level for each cultivar in each treatment.

A total of 372 OTUs were detected. The grapevines of the Chardonnay and Merlot cv showed an average of 91±15 (n = 7) and 84±19 (n = 5) OTUs respectively. As regards pest management, IPM had 93.5±17 (n = 6) OTUs, whereas organic production showed an average of 83.5±15 (n = 6). The nonparametric permutation test (after 1,000 Monte Carlo permutations) showed no significant differences between cultivars (n = 10, with all the Merlot samples being used, whereas just five Chardonnay samples at a time were taken into account, repeating the comparison test for all the combinations) or pest management types (n = 12) for each of the indices calculated above. One exception was represented by Simpson's diversity index (Fig. 2A) for the pest management category (*p*<0.05). Organic Merlot samples displayed the highest values for Shannon and Simpson diversity indices, and correspondingly the lowest values for observed species, Chao's richness and Abundance-Based Coverage estimators (Fig. 2A). Chardonnay samples from IPM vineyards displayed a contrasting picture, with converging high richness and low Simpson diversity index (Fig. 2A).

After quality filtering, the majority (92.4%) of sequences were identified at genus level. The most common endophyte belonged to the *Ralstonia* genus, which was well-represented in all treatments (Fig. 2B), and contributed up to 61% of the total endophytic community in individual samples (data not shown) with the notable exception of organic Merlot plants, where *Pseudomonas* and *Staphylococcus*, were prevalent. The *Pseudomonas* and *Burkholderia* genera were also frequent in all samples, ranging from 6 to 22%. Endophytic *Staphylococcus* was detected in eight samples, where it contributed to 1–8% of the total community, with the exception of one organic Merlot sample, where it represented the numerical majority (44%).

*The Mesorhizobium* and *Staphylococcus* bacterial taxa were enriched in plants from organic production as compared to IPM (Fig. 2B). Conversely, the *Ralstonia* genus was more abundant in IPM grapevines.

The most common bacterial genera in Merlot vines were: *Ralstonia* (35.7% of Merlot pyrotags), *Burkholderia* (13.9%), *Pseudomonas* (9.8%), *Staphylococcus* (7.2%), *Mesorhizobium* (4%), *Propionibacterium* (3%), *Dyella* (3%) and *Bacillus* (2%). In Chardonnay vines they were *Ralstonia* (44.2% of Chardonnay pyrotags), *Burkholderia* (13.3%), *Pseudomonas* (10.5%), *Mesorhizobium* (3.7%), *Propionibacterium* (3.1%) and *Dyella* (2.9%) (Fig. 2B). The OTU category significance test on pest management types showed highly significant raw p-values (*p*<0.01) for the *Mesorhizobium*, *Ralstonia*, *Burkholderia*, *Stenotrophomonas* and *Caulobacter* genera, and a significant p-value (*p*<0.05) for the *Staphylococcus* genus. When multiple inference correction for significance testing was used, the resulting p-values indicated that OTU abundances was not statistically significant, across either cultivars or pest management types. The association function revealed (after 9999 reiterations) that OTUs belonging to the *Caulobacter* and *Paracoccus* genera were significantly correlated to organic pest management (*p*<0.05). The same analysis applied to the cultivar group showed that one OTU assigned to the Enterobacteriaceae family was associated with Chardonnay and the OTUs identified as belonging to *Delftia*, *Flavobacterium* and *Massilia* genera were associated with the Merlot cultivar (*p*<0.05).

When beta-diversity was analysed using PCoA and CAP, we observed separation between endophytic communities in plants from organic and IPM vineyards (Fig. 1C and 1D), as similarly observed with B-ARISA. The permutation test to assess the significance of constraints showed pest management to be significant (*p*<0.001), while differences between endophytic communities in Merlot and Chardonnay plants were not observed. The PERMANOVA analysis applied to the Bray-Curtis dissimilarity distance confirmed that endophyte diversity is mainly affected by the different pest management types (*p* = 0.002). Moreover, the interaction between pest management and cultivar categories turned out to be significant (*p*<0.05). When PERMANOVA was applied to the UniFrac distance matrix, a significant p-value for pest management (*p*<0.01) and for interaction between pest management and cultivar categories (*p*<0.05) was still observed. PERMANOVA analysis also indicated that the differences between cultivars were significant (*p*<0.05).

### Comparison of B-ARISA and 16S rDNA gene sequencing

The permutation test based on Procrustes statistics (PROTEST) showed no association between datasets, giving a high sum of squares value (m12 = 0.71) and p = 0.91 for unconstrained multidimensional scaling, and a low (m12 = 0.15) but not significant value (p = 0.16) for constrained scaling, after 9999 reiterations. Mantel tests applied to the distance matrices used for the ordinations confirmed that the datasets obtained from B-ARISA and 16S rDNA gene sequencing were not significantly correlated.

**Figure 3:**
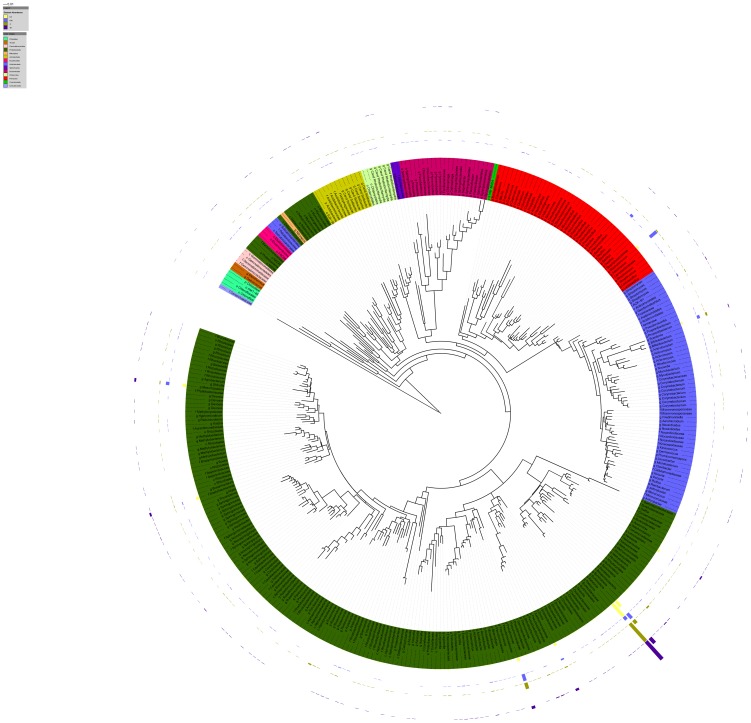
Tree of life including representative endophytic OTUs in this work. OTU colour represents phylum (see in-picture legend). Relative rarefied abundances are reported as concentric histograms. OC: Organic Chardonnay; OM: Organic Merlot; IC: IPM Chardonnay; IM: IPM Merlot.

**Figure 4:**
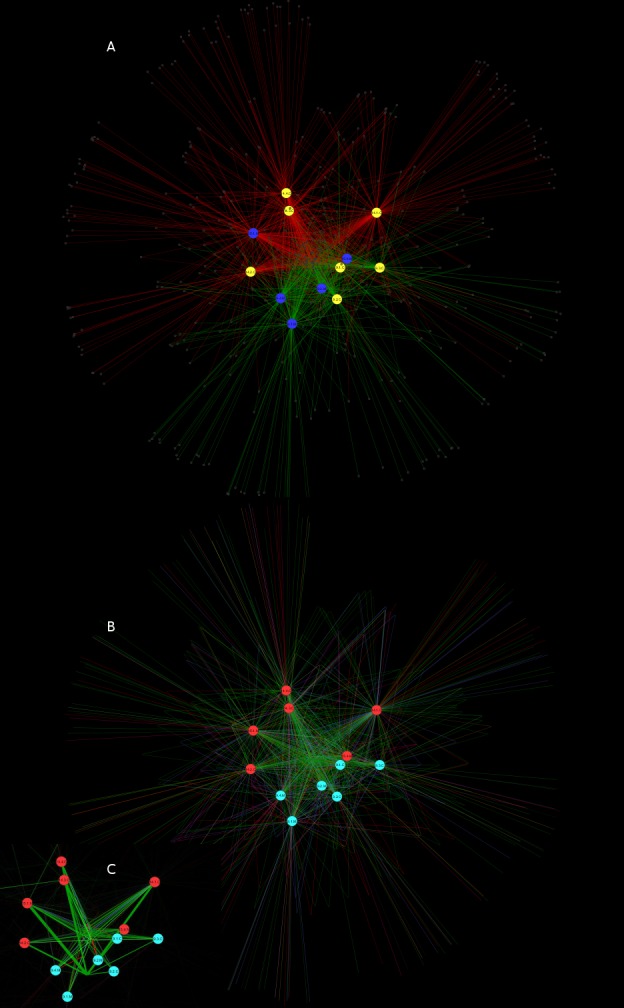
Networks representing sample/OTU interaction. In both networks edge visibility (line width and opacity) is enhanced based on eweights, to better highlight the most relevant connections. A: sample nodes are shown according to grapevine cultivar (yellow: Chardonnay; blue: Merlot), OTU nodes are white, with edges indicated according to pest management type (red: IPM; green: organic production). B: sample nodes are indicated according to pest management type (red: IPM; green: organic production), OTU nodes are white, with edges indicated according to taxonomic assignment at phylum level (colour legend as for Fig. 3). C: zoomed in view of Figure 4B, with eweight significance for edge visibility emphasised.

To unravel the complexity of sample-taxon association, we visualised the OTUs identified by pyrosequencing and their relative abundance in a tree of life (Fig. 3). This visualisation made it possible to highlight the abundance and diversity of endophytic Proteobacteria, including the highly prevalent beta-proteobacteria *Ralstonia* and *Burkholderia*. The increased relative abundance of Firmicutes in organic Merlot samples is also noticeable. Interestingly, some taxa (such as Actinobacteria and Bacteroidetes) are represented by a good number of OTUs, all with low abundance. Network visualisation of 16S rDNA sequences also highlighted the relevance of endophytic Proteobacteria in describing and shaping the network shapes and edges (Fig. 4B and 4C). In such networks (where Proteobacteria are shown in green similarly to Figure 3,), edge visibility is calibrated on eweight. The network edges (representing the connections between samples and OTUs) in the central portion of the network (the core) are largely ascribable to Proteobacteria. Other phyla are evenly distributed outside the network core and do not appear to cluster together with a specific sample. We also note that the edge-weighted spring embedded layout used here is designed to shape the network so that edge eweights are used to push similar nodes (nodes sharing OTUs) together. This layout was previously used to display genomic similarity, [62]. When distances between network nodes are optimised in this way, samples representing grapevines with identical pest management methods were grouped together (Fig. 4B and 4C), while very weak grouping or no grouping was observed when the cultivar was considered (Fig. 4A).

Network analysis eventually confirmed the results obtained using multivariate statistics, reinforcing the conclusion that pest management is the strongest driver of microbial community assembly, while the grapevine cultivar has a much weaker influence.

## Discussion

Two cultivation-independent approaches were used in this work to assess the impact of pest management (organic production *vs.* IPM) and the plant cultivar (cv Merlot *vs.* cv Chardonnay) on endophytic bacterial communities in plants of *Vitis vinifera* L., sampled in a relevant grape-growing area in Italy. We previously [12] highlighted the fact that organic production and IPM resulted in similar, yet distinguishable fungal endophytic communities. We anticipated the former finding when comparing organic production and IPM, as the synthetic fungicides used in IPM (see Table S1) could reasonably influence fungal endophytes in the plant. Here we report the less predictable observation of similar differences between bacterial communities in the same conditions (since no bactericidal antibiotics were included in the products used in the test fields).

Intrigued by the differences in the bacterial endophytic community in organic and IPM vineyards observed using B-ARISA markers, we further investigated endophytic microbial communities in a selected area by pyrosequencing the 16S rDNA gene. For this experiment we targeted all 16 samples harvested at one of the locations where sampling for B-ARISA took place, where the number of B-ARISA markers was near the average value. Pyrosequencing with new XL+ chemistry, together with a unidirectional sequencing strategy, led to the sequencing of multiple hypervariable regions of 16S rDNA on a single long read (∼800 bp), thus overcoming one of the bottlenecks associated with shorter reads. A similar approach was recently used by Pinto and colleagues [25] to sequence a much smaller fragment (∼381 bp), corresponding to the V6 hypervariable region in grapevine-associated microorganisms. We exploited the potential of long read sequencing to the fullest, amplifying a region over 700 bp long (V5–V9), and assigning a very high proportion (92.4%) of the sequences obtained to the genus level.

Although the prevalence of Proteobacteria could be inferred from the raw data, their abundance in terms of reads and OTU number is better visualised using a tree of life (Fig. 3). Comparatively, while the number of OTUs assigned to Firmicutes and Actinobacteria is also high, their relative abundance is much lower than that of Proteobacteria. The relevance of this phylum for community composition is better understood through the networks shown in Figures 4B and 4C, where the central part of the network is characterised by edges linking the OTUs assigned to Proteobacteria (shown in green) with the corresponding samples. The spatial distribution of sample nodes in these networks highlights separation according to pest management type, but not according to grape cultivar, also reinforcing our central finding that pest management is highly relevant in determining the composition and assembly of bacterial endophytic communities in the grapevine.

Comparison of bacterial endophyte community composition in organically produced and IPM vines showed analogies between the outcomes of B-ARISA and 454 pyrosequencing (compare Fig. 1A and 1B to 1C and 1D). Multilevel pattern analysis was used (as described above [54]) in order to assess the statistical significance of the relationship between species occurrence and groups of samples. Interestingly, the significant interaction between pest management and cultivar suggests that the cultivars analysed may respond differently to different types of pest management.

The presence of bacteria belonging to the *Mesorhizobium* genus (more abundant in plants from organic production where no chemical fertilisers are used) is intriguing. Mesorhizobia are known mostly for their ability to symbiotically associate with plant roots in a range of species [64], forming nodules. When associated with root nodules, they can fix nitrogen and promote plant growth [63]. Rhizobia and mesorhizobia can also transfer to the plant canopy in rice [65], where their presence has been associated with higher levels of the phytohormones indole-3-acetic acid (IAA) and gibberellins (GA_3_). Interestingly, the most common endophytic rDNA found in this study was assigned to the *Ralstonia* genus, and was present in variable but steadily high concentrations in all samples. This taxon includes, among others, the known xylem-dwelling soil-borne pathogen *R. solanacearum*. This widespread and consistent presence of *Ralstonia* is somewhat unexpected, as its presence has not previously been reported as relevant in grapevine-associated microbiota [25], and because members of this taxa are not commonly associated with an endophytic lifestyle. It is possible to speculate that the prevalence of *Ralstonia* in this work may be linked to sampling of plants at the end of their vegetative cycle, which may enrich them in more saprophytic microbiota. Further studies can be suggested, to understand how seasonal variations throughout the year affect endophytic microbiota in perennial plants. Bacteria belonging to the *Burkholderia* genus were significantly and widely present across samples. Burkholderias are common endophytes and are frequently found in root tissues [66]. They are known for the positive role played in plants (plant-growth promotion [67], protection from pathogens [68] and from stress [69], [70]). Although controversy exists regarding the possible use of burkholderias for applied uses (mostly related to the pathogenicity traits of some taxa in this genus), it is apparent that the group including pathogenic species (principally *B. mallei* and *B. pseudomallei*) is separate from that including soil isolates and plant endophytes [71], [72]. Interestingly, taxa including well-known human and animal pathogens were present among grapevine endophytes, including *Streptomyces*, *Propionibacterium*, *Roseomonas*, *Staphylococcus* and (to a lesser extent) *Stenotrophomonas* [72]. The establishment of an endophytic stage in typically animal-associated microbiota is an area where there are extensive gaps in knowledge gaps, although several studies have addressed the endophytic dwelling of enteric bacteria in vegetables [73], [74]. These key taxa, including well known animal-associated species, were especially abundant among bacterial endophytes in organic Merlot plants. Elsewhere [72], [75] we investigated the structure of the sequences classified in the *Propionibacterium*, *Staphylococcus*, *Clostridium*, and *Burkholderia* genera. We reported that in most cases, endophytic sequences were similar to those of non-pathogenic reference species, while taxa highly similar to animal-associated and animal-pathogenic species were represented by a comparatively small number of sequences [72]. In *Propionibacterium*, other findings [75] instead suggest close adaptation of the typically animal-associated bacterium *P. acnes* to the plant habitat.

The grapevine endosphere could be colonised by these taxa either from the soil or following contact with humans (during farming practices such as pruning and propagation by cutting [75]) and micro and macrofauna colonising/feeding on the plants. If they access the plant through the soil, organic fertilisation of crops may play a relevant role.

Despite being a qualitative method, B-ARISA proved to be very effective in describing the differences between the variables studied here, while pyrosequencing revealed more limited differences, although the taxa causing them could be readily identified (in contrast with B-ARISA). Bacterial ARISA markers also indicated a greater richness of OTUs in organic production as compared to IPM farms, which was not shown by 16S rDNA amplicon analysis. Accordingly, procrustes analysis showed no correlation between the beta-diversity dataset from B-ARISA and 454 pyrosequencing. To explain this effect, which is not described in previous literature (see [76] [77]), it is necessary to highlight that 454 analysis, despite the greater depth of analysis as compared to ARISA, was used on a smaller set of samples, where sample replication may be insufficient (as in the case of IPM Merlot).

The higher frequency of *Staphylococcus* (with a relative abundance of 0.76% in IPM vs. 11% in organic production) and *Bacillus* (0.1% in IPM vs. 2.8% in organic production) in the endosphere of organically produced plants suggests that some taxa may colonise them through the application of non-sterilised organic fertilisers. This speculation can only be confirmed by study of the species involved and by analysis of the endophytic isolates in organic fertilisers and the endosphere.

Overall, our findings reveal crucial details about grapevine-associated endophytic bacterial communities, pointing out some factors related to fluctuations in community composition. Interestingly, we found that organically produced plants host endophytic communities that differ from those cultivated using IPM. While this outcome was to some extent expected when fungi were taken into account [12], [78], the findings presented here show strikingly that bacterial communities are also affected by pest management.

At this stage we cannot establish how pest management affects bacterial endophytes, whether directly through treatment with chemical pesticides and fertilisers (IPM) and the use of natural plant protection products and organic fertilisers (organic production), or whether it is rather the modification of the fungal endophytic communities we described in a previous study that in turn triggers a whole-community restructuring effect. Pest management types may affect endophytic microorganisms directly or through modification of plant physiology, which may in turn have an impact on plant-associated biota by altering the expression of the plant's metabolic pathways (for example those underlying systemic resistance, tissue senescence or nutrient abundance). The mechanisms determining the response of plants and plant-associated microbial communities to external chemical stimuli are of considerable interest for agriculture and further work should focus on the response of plant endophytes to synthetic pesticides and natural plant protection methods.

### Sequence Repository

Sequence accession numbers: the pyrosequencing-generated nucleotide sequences have been deposited in the NCBI Sequence Read Archive (SRA) database under accession numbers SRR1284285- SRR1284296.

## Supporting Information

Table S1 (DOC)
