# Supplementary material for: Bacterial Endophytic Communities in the Grapevine Depend on Pest Management
Source: PLoS One. 2014 Nov 11;9(11):e112763. doi: 10.1371/journal.pone.0112763 (PMC4227848; doi:10.1371/journal.pone.0112763)
Supplement: Table S1 — (DOC) [file pone.0112763.s001.doc]

|  | In IPM only | In both Organic farming and IPM |
| --- | --- | --- |
| Treatments used | Flint (trifloxystrobin, fungicide), Melody Compact (iprovalicarb, fungicide), Topas (penconazole, fungicide), Forum 50 WP (dimetomorph, fungicide), Switch (cyprodinil and fludioxonil, fungicides), Fungiben (miclobutanyl, fungicide) | KOCIDE 3000 (copper hydroxide, fungicide), Cupron ULD (copper sulphate, fungicide), Microthiol disperses (sulphur, fungicide), Tiovit jet (sulphur, fungicide), CuSO4 (fungicide), Poltiglia Caffaro (opper sulphate, fungicide), |

**Supplementary table S1**: products, active ingredients and target microorganisms used in IPM and Organic management in the area and in the season of study
